# Trace Element Determinations in Biologicals Using Atomic Absorption Spectrometry

**DOI:** 10.6028/jres.093.073

**Published:** 1988-06-01

**Authors:** Nancy J. Miller-Ihli

**Affiliations:** U.S. Department of Agriculture, Nutrient Composition Laboratory, Beltsville, MD 20705

Atomic absorption spectrometry (AAS) has been used for many years for trace element determinations in foods and other biological materials. The Nutrient Composition Laboratory of the U.S. Department of Agriculture has both conventional commercial single element AAS Spectrometers (Perkin-Elmer Models 603 (flame) and 3030 (Zeeman, furnace)) and a multielement AAS research spectrometer (SIMAAC) [1] which can provide multielement data using either flame or graphite furnace atomization [2–3]. The SIMAAC system consists of a 300 watt xenon arc lamp, an atomizer, an echelle polychromator modified for wavelength modulation, and a PDP 11/34 minicomputer responsible for high-speed data acquisition, data processing and report generation. The SIMAAC system can simultaneously determine up to 16 elements including: Al, Ca, Co, Cr, Cu, Fe, K, Mg, Mn, Mo, Na, Ni, Pb, Sn, V, and Zn. The extended range [4] provided by the system permits simultaneous measurement of elements whose concentrations may vary by as much as 4 orders of magnitude of concentration. The SIMAAC system has been used for a wide range of applications but the maximum potential of multielement AAS can be seen using graphite furnace atomization (GFAAS) [7–9] where sub-ng/mL detection limits can be achieved using µL-sized samples.

Successful single element or multielement AAS determinations cannot be achieved without suitable sample preparation procedures. As a result, research has been conducted to evaluate the following sample preparation procedures: 1) wet ashing; 2) dry ashing; 3) microwave dissolution; and 4) direct analysis of solids.

## Wet Ashing

Wet ash sample preparation procedures are versatile and offer several advantages. First, wet ash procedures are applicable to a wide range of materials and suitable for all foodstuffs. Second, wet ash procedures are low temperature preparations and therefore provide reduced risk of loss of volatile elements. Third, high purity reagents are available for wet ash preparations and finally wet ash preparations are not labor intensive. Typically large batches of samples can be easily handled using a heating block or hot plates. A typical wet ash procedure appears in table 1. Basically, this is a HNO_3_/H_2_O_2_ digestion done using ultrapure reagents (sub-boiling distilled HNO_3_, National Bureau of Standards, Gaithersburg, MD; Perone Peroxide, DuPont, Wilmington, DE) and silanized quartz test tubes to prevent contamination. Typical wet ashing results for NBS SRM 1577a Bovine Liver and NBS SRM 1572 Citrus Leaves appear in tables 2 and 3. The average accuracy for the simultaneous multielement determination of these four elements was 100±9% and the average precision was 10% RSD.

## Dry Ashing

Dry ashing also offers several advantages as a sample preparation procedure. Dry ashing requires very few reagents, utilizes simple apparatus, requires little sample manipulation and is excellent for biological fluids. Dry ashing also shares the benefit of requiring minimal operator attention so it lends itself well to large “batch” preparations of samples. Table 4 contains a typical dry ash procedure using serum as an example. This procedure provides an optional 4-fold concentration step. Data for NBS SRM urine prepared using this procedure without the 4-fold concentration step appear in table 5. The average accuracy for the five elements determined simultaneously was 100±6% and the average precision was 9% RSD.

## Microwave Dissolution

Microwave dissolution was evaluated because of its significant time savings and potential to provide lower blanks. The closed vessel procedure evaluated provides elevated temperatures and pressures leading to more complete digestions than can be attained using conventional preparation procedures. Microwave procedures also provide the ability to use reproducible conditions making sample dissolution less of an art and more of a science. The microwave digestion procedure utilized was based on the method of Kingston and Jassie [7] (see table 6). Equipment used consisted of an MDS 81 600 watt microwave oven (CEM Corp., Indian Trail, NC) equipped with a turntable and 60 mL teflon PFA vessels with relief valves (Savillex Corp., Minnetonka, MN). Analytical data for NBS SRM 1567 Wheat Flour appear in table 7 and data for an in-house tuna quality control material are summarized in table 8. Reference values for the tuna are based on analytical results obtained using conventional wet and dry ashing preparations and atomic absorption and emission methods. Both sets of data indicate that the microwave method provides suitable results (Wheat Flour: Average accuracy 100±7%, average precision 9% RSD).

## Direct Solids Analysis

The direct analysis of solids in the graphite furnace appears to be the ultimate solution to sample preparation problems related to contamination control and preparation time. Research has been conducted using slurry preparations of a wide range of materials [8]. Slurry preparations offer several advantages over conventional wet ashing and dry ashing procedures: 1) reduced sample preparation time, 2) decreased possibility of analyte loss through premature volatilization, 3) reduced loss of analyte related to retention by insoluble residue, and 4) reduced opportunity for sample contamination. Slurry preparations also offer advantages over direct analysis of solids. First, slurries can be easily prepared and no special tools are required. In addition, any amount of the original powdered sample can be used and samples can be diluted if necessary. Finally, samples can be prepared in advance and handled easily. Slurries are typically prepared by weighing ~10 mg of finely powdered homogeneous material into a clean polypropylene tube and then diluting with 5–10 mL of 5% HNO_3_ containing 0.04% Triton X-100. Slurries are mixed well on a vortex mixer and while mixing, a 1 mL aliquot is removed and placed into an acid soaked teflon autosampler cup. Slurries are then mixed using an ultrasonic probe (RAI, Hauppauge, NY) until the autosampler withdraws an aliquot for injection into the graphite furnace. Table 9 contains analytical data from the analysis of NBS SRM Citrus Leaves and SRM 1572. Table 10 contains results for NBS SRM 1632a Coal. Average accuracies were; 100±10% (Citrus Leaves); 100±14% (Coal) and average precisions were: 11% RSD (Citrus Leaves); 6% RSD (Coal). These and similar data for more than 12 materials suggest that the slurry technique is well suited to the analysis of homogeneous powdered biological and botanical materials.

## Comparison of Methods

NBS SRM 1577a Bovine Liver was prepared in duplicate using each of the four methods discussed. Analytical results appear in table 11 with the amount of time each preparation procedure required under routine operating conditions in our laboratory. It is clear that all sample preparation methods provided accurate analytical data for the four elements determined. The microwave and slurry preparation procedures offered significant time savings over the wet ashing and dry ashing methods which were performed in the typical batch mode of operation routinely employed in the lab. Presumably, the times for wet ashing and dry ashing could be reduced somewhat but, it is clear that the microwave and slurry methods offer significant advantages with regard to sample contamination as well as speed. Additional work is needed to ascertain whether these two faster methods are well suited for as wide a range of sample matrices as conventional wet ashing and dry ashing preparation procedures are.

## Figures and Tables

**Table 1:** Wet ash digestion procedure

1.	Weigh homogenized sample (0.5–1.0 g) into a silanized quartz test tube and add 2 mL 18 megohm water
2.	Add 0.5–1.0 mL sub-boiling distilled nitric acid and heat samples (80 °C) overnight
3.	Add 1 mL ultrapure hydrogen peroxide, repeating until samples are clear
4.	Allow samples to go to dryness
5.	Dilute to a final volume of 5–15 mL

**Table 2:** SIMAAC GFAAS results for wet ashed bovine liver^a^ (µg/g, dry weight)

Element	Soln. conc. (ng/mL)	Certified value	SIMAAC value
Pb	30	0.34±0.08^b^	0.30±0.02^c^
Cr	10	0.088±0.012	0.099±0.009
Co	20	(0.18)	0.17±0.03
Mo	380	(3.4)	3.6±0.3

a Sample 1 g/10 mL.

b Values = 95% confidence level.

c Values= 1*σ*, n = 6.

**Table 3:** SIMAAC GFAAS results for wet ashed citrus leaves (µg/g, dry weight)

Element	Soln. conc. (ng/mL)	Certified value	SIMAAC value
Mn	28^a^	23±2^c^	20±2^d^
Zn	38^a^	29±2	32±2
Cu	19^a^	16.5±1.0	15.5±0.7
Pb	18^b^	13.3±2.4	14.8±2.9
Cr	120^b^	0.8±0.2	1.0±0.1
Mo	23^b^	0.17±0.09	0.19±0.05

a Sample 0.6 g/500 mL.

b Sample 0.6 g/5 mL.

c Values = 95% confidence levels.

d Values=1*σ*, n = 6.

**Table 4:** Dry ash digestion procedure

1.	Pipet 2 mL of urine into a silanized quartz test tube
2.	Add 20 µL of 2% magnesium nitrate and vortex
3.	Dry samples (or freeze and freeze-dry)
4.	Place samples in muffle furnace and heat: 100 °C 1 h; 150 °C 1 h; 200 °C 1 h; 250 °C 1 h; 480 °C overnight
5.	Dissolve ashed urine in 2 mL 5% sub-boiling distilled nitric acid

**Table 5:** SIMAAC GFAAS results for dry ashed SRM urine (ng/mL)

Element^a^	Certified value	SIMAAC value
Cu	370±30^b^	331±33^c^
Pb	109±4	98±12
Cr	85±6	86±7
Ni	(300)	294±23
Cd	88±3	84±6

a 2 mL urine dry ashed and diluted to 2 mL (5% HNO_3_).

b Values = 95% confidence levels.

c Values= 1*σ*, n = 5.

**Table 6:** Microwave preparation procedure^a^

1.	Weigh 0.15–0.20 g material into teflon PFA vessel
2.	Add 5 mL of sub-boiling distilled HNO_3_ and seal vessels
3.	Place samples on turntable and digest individually (25% power; 8 minutes)
4.	Cool vessels and remove cap
5.	Rinse cap with deionized distilled water
6.	Place uncapped vessel on hot plate (130 °C) and heat (approx. 30 minutes)
7.	Dilute to final volume (10 mL)

a Adapted from: Kingston, H. M. and Jassie, L. B., Anal. Chem. 58, 2534 (1986).

**Table 7:** Results for microwave preparation of NBS wheat flour (µg/g, dry weight)

Element	Certified value	Flame AAS value
Mn	8.5±0.5^a^	7.7±0.5^b^
Zn	10.6±1.0	11.2±0.2
Fe	18.3±1.0	18.4±3.3
Cu	2.0±0.3	1.7±0.3
K	1360.±40.	1260.±29.

a Values = 95% confidence level.

b Values= 1*σ*, n = 6.

**Table 8:** Results for microwave preparation of tuna (µg/g, dry weight)

Element	Reference value	Flame AAS value
Mn	0.43±0.08^a^	0.64±0.08^b^
Zn	13.5±0.6	13.9±0.8
Fe	30.9±1.7	32.8±1.3
Cu	1.27±0.25	1.18±0.03
Mg	1076.±59	964.±8
Na	15,976.±793	16,367.±844
K	9,113.±425	8,238.±110

a Values=95% confidence level.

b Values= 1*σ*, n = 6.

**Table 9:** SIMAAC GFAAS results far slurry preparations of NBS citrus leaves SRM 1572 (µg/g, dry weight)

Element	Certified value	SIMAAC value
Mn	23±2^a^	19+1^b^
Zn	29±2	33±4
Fe	90±10	90±15
Cu	16.5±1.0	16.1±1.0
Pb	13.3±2.4	15.3±2.5
Al	92±15	80±18

a Values = 95% confidence level.

b Values= 1*σ*, n = 5.

**Table 10:** SIMAAC GFAAS results for slurry preparations of NBS coal SRM 1632a (µg/g, dry weight)

Element	Certified value	SIMAAC value
Mn	28±2^a^	23±2^b^
Fe	11,100±200	12,100±1500
Cu	16.5±1.0	14.0±4.3
Cr	34.4±1.5	26.4±2.5
V	44±3	46±7

a Values = 95% confidence level.

b Values = 1*σ*, n = 5

**Table 11:** Comparison of methods (bovine liver, SRM 1577a)

Method	Mean concentration (µg/g)				Total time
	Mn	Zn	Fe	Cu	
Wet Ashing	10.9	125	184	159	36 h
Dry Ashing	9.7	120	190	153	24 h
Microwave	9.7	120	198	154	45 min
Slurry	10.9	131	210	153	5 min

Cert. Values:	9.9±0.8	123±8	194±20	158±7.	

## References

[1] Harnly JM, O’Haver TC, Golden B, Wolf WR (1979). Anal Chem.

[2] Miller-Ihli NJ (1985). Anal Chem.

[3] Harnly JM, Miller-Ihli NJ, O’Haver TC (1984). Spectrocim Acta.

[4] Harnly JM, O’Haver TC (1981). Anal Chem.

[5] Kingston HM, Jassie LB (1986). Anal Chem.

[6] Miller-Ihli NJ J Anal At Spectrom.

